# miRNAs in the Regulation of Cancer Immune Response: Effect of miRNAs on Cancer Immunotherapy

**DOI:** 10.3390/cancers13236145

**Published:** 2021-12-06

**Authors:** Faheem Hyder Pottoo, Ashif Iqubal, Mohammad Kashif Iqubal, Mohammed Salahuddin, Jawad Ur Rahman, Noora AlHajri, Mustafa Shehadeh

**Affiliations:** 1 Department of Pharmacology, College of Clinical Pharmacy, Imam Abdulrahman Bin Faisal University, P.O. Box 1982, Dammam 31441, Saudi Arabia; fhpottoo@iau.edu.sa; 2 Department of Pharmacology, School of Pharmaceutical Education and Research, Jamia Hamdard, New Delhi 110062, India; asifiqubal2013@gmail.com; 3 Department of Pharmacology, Delhi Pharmaceutical Sciences and Research University, New Delhi 110017, India; 4 Department of Pharmaceutics, School of Pharmaceutical Education and Research, Jamia Hamdard, New Delhi 110062, India; mkashifiqubal@ymail.com; 5 Product Development Department, Sentiss Research Centre, Sentiss Pharma Pvt Ltd., Gurugram 122001, India; 6 Department of Clinical Pharmacy Research, Institute for Research and Medical Consultations, Imam Abdulrahman Bin Faisal University, P.O. Box 1982, Dammam 31441, Saudi Arabia; msalahuddin@iau.edu.sa; 7 Department of Microbiology, College of Medicine, Imam Abdulrahman Bin Faisal University, P.O. Box 1982, Dammam 31441, Saudi Arabia; juurrahman@iau.edu.sa; 8 Mayo Clinic, Sheikh Shakhbout Medical City (SSMC), Abu Dhabi 127788, United Arab Emirates; 9 College of Medicine and Health Sciences, Khalifa University, Abu Dhabi P.O. Box 127788, United Arab Emirates

**Keywords:** miRNA mimetics, immune escape, microRNAs, immunotherapy, cancer, immune escape, immune-checkpoint molecules, tumour microenvironment

## Abstract

**Simple Summary:**

Cancer is the deadliest disease affecting humans, with more than 14 million cases and 8 million deaths. A successful therapeutic interception of cancer is urgently required. We have reviewed the immunologic involvement of various types of cancers that are well substantiated in the existing literature. Interestingly, multiple signalling pathways of the immune system could be modulated with miRNAs (noncoding RNAs of 22 nucleotides), such as miR-19a-3p, which induces macrophage polarization and the amelioration of cancer progression and metastasis. Developing a specific profile of miRNA within immune cells could also help in diagnosis and treatment. miRNA-based immune therapeutics that help to reduce cancer immune escape hold possibilities for developing cancer chemotherapy.

**Abstract:**

In the last few decades, carcinogenesis has been extensively explored and substantial research has identified immunogenic involvement in various types of cancers. As a result, immune checkpoint blockers and other immune-based therapies were developed as novel immunotherapeutic strategies. However, despite being a promising therapeutic option, immunotherapy has significant constraints such as a high cost of treatment, unpredictable toxicity, and clinical outcomes. miRNAs are non-coding, small RNAs actively involved in modulating the immune system’s multiple signalling pathways by binding to the 3′-UTR of target genes. miRNAs possess a unique advantage in modulating multiple targets of either the same or different signalling pathways. Therefore, miRNA follows a ‘one drug multiple target’ hypothesis. Attempts are made to explore the therapeutic promise of miRNAs in cancer so that it can be transported from bench to bedside for successful immunotherapeutic results. Therefore, in the current manuscript, we discussed, in detail, the mechanism and role of miRNAs in different types of cancers relating to the immune system, its diagnostic and therapeutic aspect, the effect on immune escape, immune-checkpoint molecules, and the tumour microenvironment. We have also discussed the existing limitations, clinical success and the prospective use of miRNAs in cancer.

## 1. Introduction

According to the World Cancer report 2020, Cancer is the 1st –2nd cause of premature death in 134 out of 183 countries and ranks as the 3rd–4th cause in 45 of the remaining countries. *(WHO World cancer report 2020)*. It has been estimated that by 2030, cancer prevalence might increase to around 26 million and will lead to 17 million deaths [1]. Around 1.9 million people were diagnosed with cancers in the USA alone, which were statistically expected to lead to 0.61M deaths in 2021 [2,3]. The disease presents serious threats and challenges due to its ability to cause tissue damage and difficulties in diagnosis and prospective treatment [4,5,6]. Cancer leads to a devasting effect on patients’ quality of life and leads to a devasting effect on patients’ quality of life and imposes a serious economic burden on nations [1]. Considering the aetiology of tumorigenesis, a successive mutation in response to internal as well as external stimuli in genes or DNA leads to abnormal changes in the cell functions and gradually causes an imbalance between cell division and apoptosis, leading to the initiation and progression of tumorigenesis. Various factors such as smoking (carcinogen), environmentally hazardous chemicals, genetic factors, viruses, bacteria, and radiation influence the cytoplasm and nucleus, ultimately causing genetic disorders and gene mutations, leading to cancer [7,8,9,10].

Currently, cancer treatment relies on various factors such as phenotype, tumour size, tumor volume, localization, stage of spread, mutagenic analysis, coexisting disease condition, the physical status of patients and any possible side of anti-cancer drugs. It also depends on treatment approaches of combining two or more therapies such as chemotherapy, radiotherapy, hormone therapy, etc. However, these approaches are not sufficient to treat cancer in the advanced stage, ultimately leading to metastasis and death [11]. The available therapeutic regimen leads to serious adverse effects with economic burden and poor patients’ quality of life. Studies have shown the involvement of various altered signalling molecules and pathways in the pathogenesis of tumors. In the past, several significant achievements in the field of research and development of anti-cancer drugs and their derivatives have been made, especially in treatment and diagnosis [12,13,14,15]. Apart from the role of drugs in the treatment of cancer, a timely diagnosis also plays a pivotal role in the management of various types of tumours. With the advancement in techniques, various cellular and molecular diagnostic markers have been developed, but cancer’s complexity presents a significant challenge in determining the accurate diagnosis [16,17]. The “omics” revolution has highlighted the complex nature of cancer signalling pathways, types of molecules, and cancer alterations, due to the greater capacity of noncoding MicroRNAs (miRNAs) to alter expression. Various studies have reported miRNAs’ ability to express themselves differentially in multiple types of cancer [18,19,20].

The modulation of miRNA expression is reported to be related to oncogenic miRNA inhibition and the substitution of tumour-suppressive miRNAs and, hence, have been found to be a perfect tool for cancer therapy improvement. The association of miRNA expressions with cancer and other problems has attracted interest in developing a biomarker for diagnosis, detection, and treatment [21,22,23]. miRNAs have been reported to play a protective role as well as a pathogenic role in carcinogenesis. Therefore, it becomes important to selectively identify the exact role of miRNA and, accordingly, miRNA-based therapeutics can be designed and developed. Research is currently underway concerning the development of miRNA-based diagnostic and therapeutic tools for the timely management and treatment of cancer. Thus, the present review mainly focuses on extracellular miRNA’s biogenesis, functions, and role as a biomarker in diagnosing and treating various cancers.

## 2. miRNAs—Biogenesis, Biochemistry and Functions

It has been reported that DNA acts as an immediate precursor to miRNAs. Initially, miRNAs (pri-miRNAs) are primary are produced and later converted into precursor miRNAs (pre-miRNAs) then mature miRNAs [17,24]. This process begins with the biogenesis of canonical miRNAs in the nucleus due to the activation of polymerase II, leading to primary miRNA transcript (pri-miRNAs) formation outside of the genome non-coding fraction. These primary miRNAs are trims that are processed by a microprocessor with an endonuclease III enzyme and binding protein (DiGeorge syndrome), liberating pre-miRNAs hairpin-like structure [25,26,27]. Within pri-miRNA, DGCR8 binds to pri-miRNA at N6- methyladenylated GGAC and other motifs, whereas Drosha cleaves to the pri-miRNA duplex at the base of the hairpin structure of pri-miRNA to produce precursor miRNA (pre-miRNA). These generated pre-miRNAs are carried to the cytoplasm by Exportin 5 and Ran GTPase complex and then processed by RNase III endonuclease. The direction of the miRNA strand determines the name of the miRNA. Like 5P from the 5′ end of and 3p from the 3′ end of the pre-miRNA hairpin [28,29,30]. In the maturation stage, Enzyme RNase III endonuclease in the cytoplasm, combined with TRBP and PACT proteins, break it to produce a mature miRNA duplex. Then, the mature single-stranded miRNA (generally lower 5′ stability), along with protein (Argonaute family) Ago, are loaded together with RNA, inducing a silencing complex after which it binds to 3′ UTRs (mRNA targets), causing decapping and deadenylation of the mRNA transcript, thus inhibiting various physiological functions. The unloaded miRNA strand is unwound from the guide strand, referred to as the passenger strand [31,32]. After coupling RISC with miRNA, it silences the posttranscriptional modification by tethering to become partially compatible with mRNA within the 3’untranslated region found in the UTR. Binding at seed sites or at the non-canonical seedless site of the miRNA-mRNA linkage network is complex as one miRNA affects multiple genes. One mRNA might be targeted by numerous related or unrelated miRNAs [33,34]. It has been reported that one miRNA complex can target almost two hundred genes of variable functions [35]. About 60% of human genes can be targeted by these miRNAs, once their corresponding mRNAs targets are regulated by miRNA, influencing various pathways, including cancer [36,37].

Since the identification of tumour-suppressive miRNA gene miR-15a/miR-16-1, the functional significance of miRNAs has been realized, and nearly 2500 miRNAs are recorded in the database of miRBase [38]. These miRNAs are small, non-coding RNA molecules of 22nt (average) in size and are responsible for gene regulation [39], for various biological processes and carcinogenesis [11,40]. miRNAs act as central regulators of gene expression by acting on mRNA or its translation. They are crucial in remedying various human ailments and cancer [41].

Several researchers have reported the location of most miRNAs’ genes at cancer-related genomic regions known as fragile sites. The expression data of cancer cells shows that these miRNAs are expressed by rule rather than the exception [42,43]. miRNAs play an important role and have been reported to be involved in lung, colon, breast, and other cancers. Most researchers have reported that miRNAs exhibit biochemical and molecular effects by targeting multiple mRNAs, and a few of these mRNAs are present in the cellular pathway. A few studies have reported that these mRNA were redundant and repressed the same mRNA target. In the animal model, the link between miRNA and cancer is detected by overexpressing miRNA features [43,44].

mRNAs functions have been categorized as homeostatic regulation of gene expression and cellular responses robustness. These two factors play important functions by fine-tuning translation and cell-fate decisions through complex reciprocal negative-feedback loops. Stress requires a robust response from miRNA, which may act as switches and help adapt to microenvironment changes [11,45]. This function can be best observed in glioblastoma, where glucose level reduction causes a reduction of miR-451 levels. When sufficient energy is available, the elevated level of miR-451 suppresses the cell signalling pathway and stimulates the activation of the mammalian target of rapamycin and cell proliferation [46,47]. Every tumour has its own miRNA sign, which can be helpful in differentiating these from other healthy tissues and in identifying the different types of cancer. Based on these miRNA signs, most cancers can be divided into prognostic groups. The functions of amplification or deletion, methylation, and transcription factors are responsible for altering the MicroRNA expression [47,48].

### 2.1. Oncogenic miRNAs

Many miRNAs have been reported to be involved in various biological functions, including gene regulation. Variations in the expression of these miRNAs were found to be responsible for causing different diseases, including cancer. Specifically, changes in miRNA levels with oncogenic or cancer suppression properties are related to carcinogenesis, metastasis, and the anticancer responses to drugs and therapies. Intergenic regions between genes possess several miRNA sequences. In some cases, miRNAs that are located close to each other are controlled by the same promoter and play an important role in more than one cancer by acting through various pathways. Their expression results in the formation of a multiple–looped polycistronic sequence from which multiple mature miRNA are synthesized [49,50,51,52]. These miRNAs are termed as “oncomirs” or “oncomiR” and regulate the progression of the tumour (Table 1). For example, miR-21 acts as an oncogene and suppresses TPM1 and PDCD4 (tumor genes) by downregulation in high proliferated breast cancer [49,53,54]. Cancer is caused due to imbalances in the cell cycle. Different mechanisms such as cell cycle regulation, detection, and damage repair control these imbalances in regulatory pathways. Cyclins and cyclin-dependent kinases (CDKs) are regulatory proteins and are responsible for cell progression in the cell cycle [24,53].

Several studies support the function of miRNA as tumour suppressors [55], e.g., miR-34 represses tumour progression through epithelial-mesenchymal transition (EMT) via EMT-transcription factors when dysregulated via the synergistic effect of the p53 tumor suppressor gene and some important signal pathways. Several *invitro* studies on various cancer cell lines found that through different signalling pathways downregulation of the family of miR-34 can lead to colon, prostate, lung, osteosarcoma, and other types of cancers [54,55,56,57,58,59]. Similarly, Pichiorri et al. found the overexpression of miR-106b∼25 cluster, miR-181a and b and miR-21 in MM cell lines leading to the tumour compared to normal cell controls thus acting as an oncogene when two miRNA cluster. In contrast, miRNA-106b, miRNA-181a and b were found to be deregulated, thus acting as tumour suppressors [58].

### 2.2. miRNAs as Biomarkers

miRNAs can function as biomarkers to identify various diseases, be found in plasma, serum, semen, and other biological fluids, and even be stable in degrading conditions [81,82,83]. There are two clusters or populations of circulating miRNAs, one cluster is present in vesicles and the other is linked with proteins. Their association with these two locations has been contradicted by researchers who hypothesize that most circulating miRNAs are exosome miRNAs [41,84,85].

The ceramide-dependent pathway is involved in the release of exosomal miRNAs in response to injury or even the cell’s death. It thereby produces regulatory effects in target cells [86]. Interleukin-4 and docosahexaenoic acid regulate the secretion of miRNAs via exosomes. In breast cancer, interleukin-4 activation leads to the secretion of oncogenic miRNAs. It stimulates cancer cell invasiveness whereas, docosahexaenoic acid exhibits anticancer effects by stimulating the exosomal miRNA secretion, thereby producing tumour angiogenesis [83,87,88]. Recent studies have reported exosome miRNAs’, such as miR-105, role in the metastatic breast cancer cell, miR-21-3p from umbilical cord blood, in destructing the barrier function, metastasis and the migration of fibroblasts, leading to the angiogenic action by endothelial cells. miRNAs such as miR-342–3p and miR-1246 from oral cancer are reported to be responsible for inducing metastasis in metastatic cancer cell line [83,89,90,91].

The identification of serum levels of cancer patients was performed using seven miRNAs and found a significant reduction in serum levels of advanced stage astrocytoma’s patients and helps in precisely distinguishing between normal and cancer patients [92,93]. Thus, circulating miRNAs possess a differential expression ability and can act as a potential tool for screening cancer patients without harm. During the early stage, progression and even after the metastasis stage, there abnormal changes in miRNAs levels occur. Therefore, these miRNAs may act as potential biomarkers to differentiate tumours, formulating treatment strategy and prove helpful in the outcomes of the treatment [94,95,96]. miRNAs produce pleiotropic effects and are helpful in the diagnosis and prognosis of patient evaluations (Table 2). Therefore, these miRNAs are recognized as chemical messenger and regulate cellular communications [97,98,99].

In various published reports, a correlation between types of breast cancer was identified relating to the overexpression of miR-21, miR-155 and miR-106a and under-expression of miR-335, miR-126 & miR-199a with the non-tumour samples [83,100,101]. Studies have shown that miR-21 expression is not only able to distinguish between a normal and breast cancer but can also help to differentiate a metastatic patient from a patient with locoregional reoccurrence [102]. A similar study reported the overexpression of miR-21, miR-155, miR-10b in plasma samples in breast cancer patients as compared to normal control. Additionally, the level of the miRNA mentioned above complex was reduced after different treatment approaches [103,104].

Multiple myeloma is a cancer of the bone marrow, affecting various parts of the body. Several studies reported that the association of the disease progression or stages involves circulating miRNA [58], e.g., the combination of miRNA-720 and -1308 can be helpful in distinguishing a Healthy Donor from Multiple myeloma patients. Moreover, the association of miRNA-1246 and MiRNA-1308 can be helpful in determining monoclonal gammopathy of undetermined significance (MGUS) from the MM condition [105]. Xu et al. performed a meta-analysis of the 15 publications and found 7 miRNAs related to MM patients’ survival and prognosis. The results of the study revealed that reduced miR-744, miR-16, miR-15a, miR-25 and let-7e expression causes minimal overall survival in MM patients while the downregulation of miR-15a, miR-25 and miR-16, and upregulation of miR-92a were associated with shorter progression-free survival [106].

## 3. Innate and Adaptive Immunity

The immune system refers to various biochemical processes that protect multiple organs from pathogenic infections, toxins, and cancerous cells [108]. Apart from the presence of different immune organs such as the thymus and spleen, the immune system is broadly studied under the heading: innate and adaptive immunity, where innate immunity offers the first line of defence [109]. Innate immunity is considered antigen-independent, affording protection within a short period but devoid of immunogenic memory [110]. However, adaptive immunity is antigen-dependent and, therefore, takes time to exert a protective effect [111]. Additionally, adaptive immunity involves immunogenic memory and therefore, upon the repeated exposure of the antigen, a more efficient and rapid immunogenic response is observed [112]. Hence, innate and adaptive immunity are complementary immunogenic mechanisms whereby a defect in one system affects the other.

Innate immunity primarily functions to recruit various immune cells (phagocytes such as neutrophils and macrophages, natural killer cells (NK cells), dendritic cells, basophils, eosinophils, and mast cells) at the site of infection via the production of various cytokines and chemokines [111]. Tumour Necrosis Factor-alpha (TNF-α), interleukin-6 (IL-6), interleukin-1 (IL-1), etc., are some of the commonly observed cytokines that help in clearing the pathogens, but their dysregulated production results in inflammation [113]. Furthermore, the immune system also activates the adaptive immunogenic response via a regulation of antigen-presenting cells (APCs). Phagocytes (neutrophils and macrophages) exhibit bactericidal properties whereby neutrophils are short-lived, whereas macrophages are long-lived and, along with dendritic cells (DCs), additionally assist in the presentation of antigen to the T-cells [112]. Natural killer cells are an important component of innate immunity and are actively involved in the anti-cancer effect by killing tumour cells [114]. NK cells release perforin granzymes and release interferon-gamma (INF-y) that induces apoptosis, leads to the mobilization of APCs, and exhibit the anti-tumour effect [115].

Adaptive immunity works alongside innate immunity, and its function becomes important when innate immunity is ineffective or dysfunctional [111]. Antigen-specific T-cells are the primary component of adaptive immunity that become activated and proliferated in response to APCs and B cells. T cells are the derivative of hematopoietic stem cells (HPSc) and become matured in the thymus. T-cells possess T-cell receptors (TCRs) that can bind with the antigens in response to the signals from APCs, DCs, macrophages or B cells and eliminate them. APCs are immune cells that have a portentous component known as major histocompatibility complex I or II (MHC-I or II) on its surface [112]. APCs digest the antigens and display appropriate protein fragments bounded to the MHC, identified by the T-cells and activated. Activated T cells further regulate the production of cytotoxic T cells, i.e., CD8+ cells or T-helper cells (Th cells), i.e., CD4+ cells.

On the one hand, CD8+ cells destroy the infected cells or tumour cells. On the other hand, CD4+ cells are devoid of a direct cytotoxic effect but release certain cytokines that regulate the immunogenic response [112]. Additionally, Th1, Th2 and Th3 are some of the common Th cells, and Th1 regulates the production of INF-y and modulates the differentiation of B cells to produce antibodies; Th2 cells regulate the release of IL-4, IL-5, IL-13 and regulate immunoglobulin E development, whereas Th-17 regulates the formation of the IL-17 family [116]. In addition to the subtypes of T helper cells, T regulatory (T reg) cells (a subset of T-helper cells) are also an important component of adaptive immunity and ideally act as immunosuppressors and thus, control the hyperimmune responses and play an important role in killing cancerous cells [116].

## 4. Cancer Immune Escape

As is well known, the immune response in the anticancer effect is complex and consists of multiple immunogenic events whereby the production and release of cancer-associated antigens (CAAs), the processing of APCs, priming, the activation of T-cells and the cytotoxic effect of effectors are important steps [117]. Apart from this, CD8+ cytotoxic T cells (CTL) and CD4+ helper T (Th)1 cells have been reported to curb tumour progression via the release of cytotoxins and production of INF-γ [118]. These findings unequivocally demonstrated that immune editing could be used to manage and treat various types of cancers [117]. Therefore, attempts have been made to stimulate immune editing whereby the innate or adaptive immune system recognizes the tumour or presence of tumour antigens cells and destroys them. Thus, an appropriate balance between immune-inhibitory and immune-stimulatory factors is pivotal for different immune-pharmacotherapeutics’ significant anti-cancer effect. However, while using immunoediting, cancer immune evasion was found to be a major challenge for oncologists as it restricts the favourable clinical outcome. The role of regulatory immune cells, alteration in APCs, immune inhibitory cytokines and immune checkpoints are considered major contributors to the cancer-immune escape [119]. Treg are important immunosuppressive CD^+^ T cells, and the hyperactivation of Treg has been reported to mitigate the anti-tumour effect of effector cells via the production of TGF-β and IL-10 [120]. Much of the published evidence has shown that Tregs, derived from tumour cells, exhibit an increased tumour evasion effect as compared to naturally existing Tregs [121]. Additionally, Treg downregulates the level of IL-2 and upregulates the level of programmed death-1 (PD-L1), T-cell immunoglobulin mucin-3 (TIM-3), and cytotoxic T-lymphocyte-associated protein 4 (CTLA-4) etc. and stimulates cancer immune escape [122]. As it is well documented, the cytotoxic potential of effector cells depends on antigens’ expression on cancer cells. However, cancer cells cause alterations in the antigen processing machinery, resulting in the loss of these tumour-associated antigens (TAAs) and cause cancer immune escape [123]. Further, alterations in the major histocompatibility complex-1 and transporters of antigens processing mutation restrict the identification of cancer cells and, thus, promote cancer immune escape [124]. Apart from the factors mentioned above that are responsible for cancer immune escape, the role of inhibitory cytokines and immune checkpoints cannot be ignored. Numerous published studies have confirmed the role of Transforming growth factor-beta (TGF-β) in altering the TME. TGF-β, IL-8, TNF-α and IL-6, Colony-stimulating factor (CSF)-1, IFNs cause an alteration in Treg cell diffraction and stimulate a Treg-mediated increased expression of PD-L-1, inhibiting the anti-tumour effect of cancer-effector cells and promoting cancer immune escape [125]. Nevertheless, the overexpression of immune checkpoints and their ligand, such as PD-L1, plays a critical role in tumor immune evasion by stimulating the production of defective tumour-infiltrating lymphocytes that eventually become devoid of tumour-killing properties [126] (Table 3).

## 5. Immuno-miRNAs: Central Regulators of Immunity

**Figure 1:**
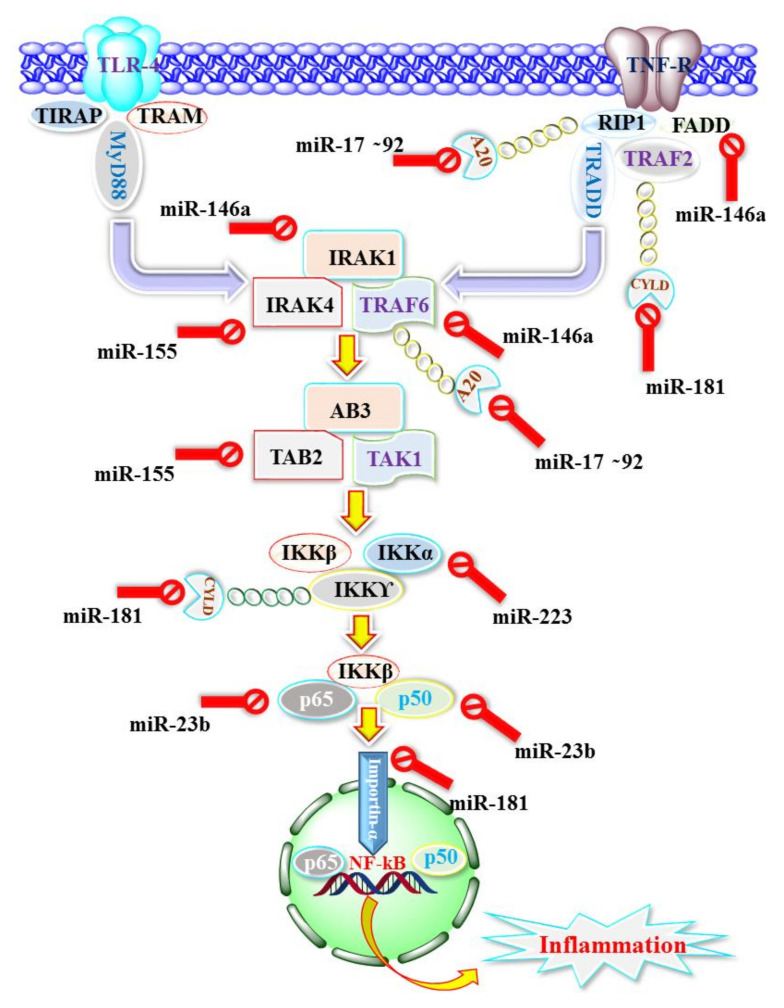
Showing the involvement of miRNAs as the central regulator of immunity via NF-kB pathway. TIRAP, TRAM, TLR4, TNF-R, RIP1, FADD, TRAF-2, TRADD, IRAK, TAB-2, TGF-β-activated kinase 1/MAP3K7-binding protein 2; TAK, IKK, and CYLD.

In recent years, various miRNAs have been explored for their pivotal role in regulating innate and adaptive immunity’s immunogenic key processes related to cancer. These miRNAs and miRNA clusters were crystallized, and attempts were made to understand the signalling pathway and the modulation of various genes related to the immune system [84,128]. Among the various signalling pathways, the nuclear factor kappa-light-chain-enhancer of activated B cells (NF-kB) was found to be a major player in the immune system (innate and adaptive immunity) during tumorigenesis (Figure 1). Under normal physiological conditions, NF-kB remains in the cytoplasm in association with its negative regulator, ikB, which in turn is regulated by the IκB kinase (IKK) complex [129]. In response to the oncogenic stimulus, the level of IKK reduces, leading to the phosphorylation of iKB and the activation of NF-kB, thus triggering its nuclear translocation and macrophage activation, which ultimately results in an inflammatory state [130]. Furthermore, MiR-223 is reported to increase the level of IKK, which in turn suppresses the ikB and keeps the NF-kB sequestered in the cytoplasm, leading to the mitigation of inflammation [131]. Apart from miR-223, various other miRNAs play a critical role in regulating NF-kB mediated immunogenic status (Figure 1). Moreover, miR-23-27-24 clusters have been reported to regulate the immunogenic status. The positive transcription of this cluster is induced by the myeloid transcription factor (MTF) known as PU.1. In contrast, it is negatively regulated by the lymphoid transcription factor (LTF) such as paired Box 5 (PAX5), Early B-cell Factor-1 (EBF1) and E2A [132]. This miR cluster has been reported to alter lymphoid cells’ differentiation and promote the lineage commitment of myeloid lineage [133]. Additionally, these clusters negatively impact T cells and repress the Th2 immunity via interaction with the IL-4 signalling pathway [134]. Apart from the negative regulation of Th2, miR-24 has been reported to promote the differentiation of Th1, T reg cells and Th17 whereas miR-23 and 27 restrained the functions of Th1 and Treg/Th17 differentiation [134]. Studies have also shown the involvement of these clusters in the suppression of CD8^+^ and the stimulation of forkhead box P3 (FoxP3) via the inhibition of NF-kB under the influence of TGF-β as well as via TGF-β mediated cMyc expression [135]. Furthermore, miR17-92 clusters are also important miRs, present on chromosome 13 and accountable for the transcription of six polycistronic miRNAs, i.e., miR-17, miR-92, miR-19b, miR-81a, miR-19a, miR-20a and [136]. This cluster of miRs is pro-inflammatory and plays a pivotal role in T-cell mediated inflammation. The activation of NF-kB is one such cause, which in turn regulates the Janus kinase signal transducer and activator of transcription (JAK-STAT) signalling pathway and stimulates the Th1-mediated INF_ϒ_ response [127]. Furthermore, miR17-92 clusters amplify the production of IL-4, IL-5, IL-13, and the development of B cells and modulation of the Inducible T-cell costimulator-phosphatidylinositol-3-kinase (ICOS-PI3K) pathway [39]. Recently emerged evidence has shown that miR17-92 clusters cause macrophage differentiation suppression, by targeting hypoxia-inducible factor-alpha (HIF-α) [137]. Other important miRs include MiR-146a and 155, which are found in abundance in innate and adaptive immunogenic cells [138]. Interestingly, miR-146a exerts an anti-inflammatory effect, whereas miR-155 exhibits a pro-inflammatory effect. The pro-inflammatory effect of miR-146a is exhibited by targeting interleukin-1 receptor-associated kinase 1 or 2 (IRAKI 1 or 2), myeloid differentiation primary response 88 (Myd88), tumour necrosis factor receptor (TNFR)-associated factor6 (TRAF6), Toll-like receptors (TLRs) and NOTCH1. The anti-inflammatory effect in the innate immune cells is exhibited by targeting TNF-α and IL-6, whereas in the adaptive immunogenic cells, it is caused via Th1 differentiation [139]. Furthermore, the anti-inflammatory effect of miR-146a is strengthened by findings where miR-146a deficient mice showed hyper inflammation and the activation of CD8^+^ and CD4^+^ cells [140]. MiR-155, on the other hand, showed a pro-inflammatory effect via the modulation of INF-_ϒ_ and production as well as activation of CD8^+^ and CD4^+^ cells [141]. The pro-inflammatory effect of miR-155 is further strengthened by the findings where the deficiency of miR-155 causes impairment of Th1 and Th2 activities [142]. Thus, based on these facts, it can be concluded that miR-146a and 155 form ‘ying-yang’ and regulate both innate as well as adaptive immunogenic cells. Both MiR-181 and miR-223 are important anti-inflammatory miRs, and miR-223 is considered as one of the classical innate miRNAs. Earlier, this miR was thought to be myeloid-specific (expressed only in granulocytes cells), but studies have now demonstrated its expression in monocytes and as well as in the macrophages [138]. During monocyte and macrophage differentiation, the level of miR-223 decreases, whereas its level increases that upregulate the activity of IKK-α. The upregulation of IKK-α prevents the phosphorylation of ikB and its activation and the nuclear translocation of NF-kB, leading to the amelioration of TNF-α, IL-6, IL-1β production [143]. However, the activation of the macrophage, which eventually activates the NF-kB signalling pathway, has been reported to downregulate the expression level of miR-223. Thus, miR-223 can be considered as multifactorial in the function whereby it regulates NF-kB, TLRs, NOD-, LRR- and pyrin domain-containing protein 3 (NLRP3) and mitogen-activated protein kinase (MAPKs) signalling pathway and exhibits a potent anti-inflammatory effect [143]. In addition to miR-223, miR-181 is another potent anti-inflammatory miR-NA that exhibits an anti-inflammatory effect in innate and adaptive immunogenic cells. Studies have shown that miR-181 mediates the suppression of tyrosine as well as extracellular signal-regulated kinase (ERK) phosphatases, Th1 differentiation and Treg differentiation by targeting the Smad7/TGF-β signalling pathway [144]. Additionally, direct targeting of miR-181 has been reported to modulate CCAAT/enhancer-binding protein alpha (C/EBP-α) and IL-1α in the macrophage and the dendritic cells, respectively [145]. Thus, miR-181 promotes M2 polarization in the macrophage, whereas in the dendritic cell, it acts on cFOS (pro-inflammatory transcription factors) [146].

## 6. Immune Cell Pathways Regulated by miRNAs in Cancer

In recent years, numerous published studies have highlighted the relationship between the immune system and tumorigenesis. In lieu of this, miRNAs have been reported to play a central role in regulating various immunogenic cells that directly affect the tumorigenesis event.

### 6.1. Regulation of Monocytes and Macrophages by miRNA

The direct impact of various monocytes has been reported on the maturation, proliferation, and differentiation of monocytes. Furthermore, MiR17-5p, miR-106p, and miR-20a have been reported to involve monocytes’ maturation via the modulation of the expression level of acute myeloid leukaemia-1 (AML-1), a well-known transcription factor [147]. Overexpression of this transcription factor in response to the aforementioned miRs stimulates the M-CSF transcription receptor that eventually causes monocytic differentiation and maturation [148]. In addition, miR-17-92 and miR-21, miR-424 also modulate monocytes’ differentiation via acting on MAPKs, JAK-STAT and TGF-β signalling pathways [149]. Macrophages (M1 and M2 exhibit the opposite effect on tumorigenesis, where M1 macrophages are activated via Toll-like receptors (TLRs) and INF-y leads to the production of pro-inflammatory cytokines such as TNF-α, IL-6, 1L-12, and IL-23 [150]. M1 macrophage also regulates T helper cells’ activity and thus is involved in the anti-tumor effect. The M2 macrophage, on the other hand, is activated in response to IL-4 and IL-13, releases IL-10 and TGF-β and is involved in tumorigenesis pathways [150]. Both miR-21 and miR-146a have been reported to attenuate the activation of M1 macrophage via TLR/NF-kB pathways, whereas miR-155 causes M2 macrophage activation via the regulation of cytokine signalling-1 supression1 [151].

### 6.2. Regulation of Natural Killer Cells (NK Cells) by miRNA

NK cells are an important component of innate immunity and are directly involved in the tumour-killing activity and the release of INF-y. Although miR-150 regulates its maturation, miR-181 regulates its development via CD3^+^ cells and Nemo-like kinase signalling molecules, miR-155 stimulates the production of INF-y and miR-29 mitigates its production [152]. As we have already discussed, NK cells exhibit cytotoxic activity against tumour cells via perforin and granzyme B and miR-30e, miR-378 have been reported to stimulate these proteins’ release as mentioned earlier [153].

### 6.3. Regulation of T Helper Cells and Cytotoxic T Cells by miRNA

T cells are the main component in adaptive immunity and miRs have been reported to regulate these cells’ activity. Furthermore, miR-155 stimulates the CD+ T cells differentiation towards Th2, whereas miR-17 and miR-19b regulate the activity of Th1 cells and increases the production of INF-y [136]. Additionally, Th17 is another important subtype of T helper cells that is responsible for the production of IL-17 [154]. Th17 has been reported to exhibit an antitumor effect via stimulating the activity of INF-y, CD8+ and NK cells in the TME [154]. Both miR-326 and miR-181c have been reported to regulate the development of Th17 by acting on Ets-1 and Smad7, respectively [155]. Furthermore, the increased level and activity of cytotoxic T cells (CD8+ cells) is responsible for the tumour-killing activity. It has been reported that miR-17-92 clusters that are present in the CD8+ cells stimulate the release of INF-y and enhance cytotoxicity towards tumour cells [156]. While miR-155, miR-21, and miR-30b promote the proliferation and cytotoxic activity of CD8+ cells via targeting cytokine signalling 1 (SOCS-1) suppression and Dual Specificity Phosphatase-10 (DUSP-10), miR-29 negatively regulates the activity of CD8+ cells and reduce the expression level of mRNA responsible for producing INF-y [157].

### 6.4. Regulation of Immune-Checkpoint Molecules by miRNA

Programmed Cell Death Protein 1 (PD-1) and its ligand, which is PD-L1, have been reported to play a pivotal role in tumorigenesis via T cell activation. PD-1 is extensively expressed on NK cells, T cells, macrophages, and on the monocytes and thus, inhibits an innate and adaptive immunogenic response [158]. PD-L-1 on the one hand is extensively expressed on tumour cell surfaces. When it binds with the PD-1, it activates tumour survival pathways, contributes to tumour immune escape, and stimulates tumour proliferation [159]. Various miRNAs have been reported to interact and modulate the activity of PD-1 and PD-L-1. It has been found that miR-155 increases PD-L-1 expression, whereas miR-34a has been reported to inhibit the expression of PD-L-1 [160]. Similarly, miR 33a, miR-21 and miR-873 have been reported to negatively regulate the expression of PD-L-1, whereas miR-146a stimulates the expression of PD-L-1 [161] (Table 4).

## 7. Effect of miRNAs on Cancer Immunotherapy

Initially, miRNAs were used as biomarkers in cancer diagnosis, prognosis, and response of pharmacotherapeutics [185]. However, it was later found that these miRNAs possess intense modulatory properties against tumorigenesis and hence, can be used as a therapeutic tool as well as a target against various types of cancers [186]. We have also discussed the way that miRNA modulates immunogenic cells in the microenvironment of tumours. Therefore, miRNA emerges as a potential future therapeutic tool against cancer treatment [187]. Currently, two broad categories of microRNA, i.e., miRNA mimics and miRNA inhibitory therapy, are being explored to determine their role as an immunotherapeutic agent in managing and treating cancer. More specifically, miRNA mimics functions to restore or promote the miRNAs that exhibit tumour-suppressing or antitumor effects whereas miRNA antagonists act against those miRNAs that stimulate tumorigenesis [187].

Additionally, miR-43a acts as an inhibitor of PD-L-1, reduces the expression of PD-L-1 mRNA, exhibits an anti-tumor effect, and mimics MRX34 enters a clinical trial to explore its antitumor effect [188]. Aside from reducing the expression of PD-L-1MRX34 also stimulate CD8+ cells tumour infiltration in non-small-cell-lungs carcinoma [188]. Further studies have shown an enhanced antitumor effect of radiotherapy when combined with MRX34 [188]. The administration of mimic MiR-124 also possesses an anti-tumour effect, and hence, miR-124 increases the level of INF-y, IL-2, and TNF-α [180].

**Figure 2:**
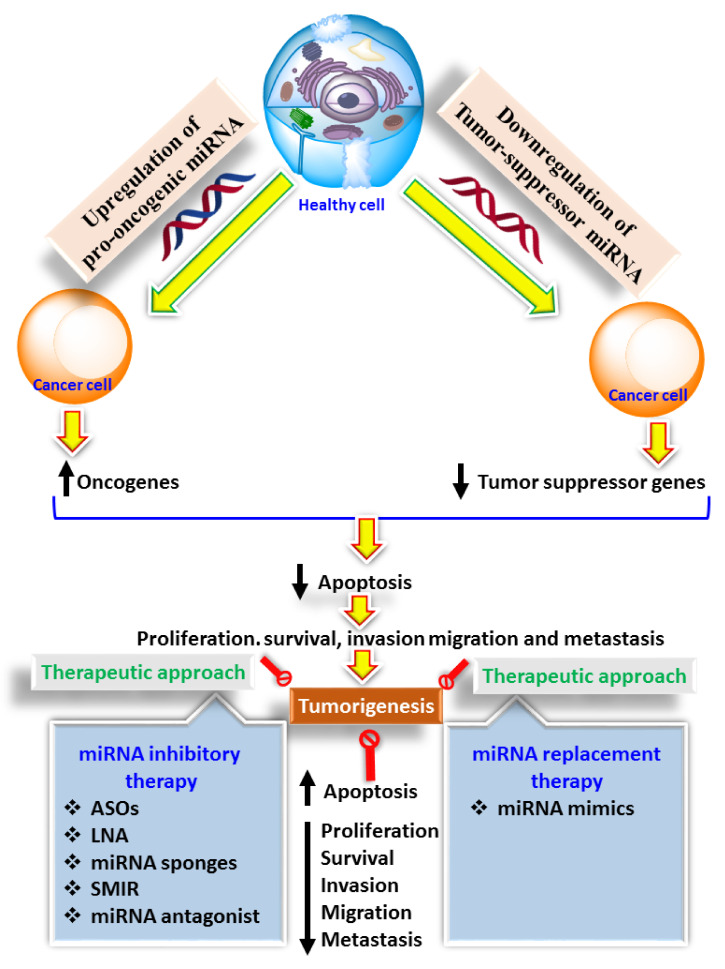
Showing the immunotherapeutic approach of miRNA in cancer.

Apart from miRNA mimics, miRNA inhibitors have also been explored as potential immunotherapeutic agents. As we know, miRNAs with pro-oncogenic properties on the tumour cells and lead to their inhibition present a potential immunotherapeutic approach. miRNA inhibitors include locked nucleic acid (LNA), anti-sense anti-miRNA oligonucleotides (AMOs), miRNA sponges, anti-miRNAs, miRNAs masks and small molecule inhibitors of miRNAs as shown in Figure 2. AMOs are single-stranded nucleotides (17-22) and act as a competitive inhibitor of pro-oncogenic miRNAs [187]. LNA is also a type of AMOs, but in AMOs, ribose sugar is modified by forming a methyl bridge and this specific modification results in thermal stability, higher water solubility and stabile in the metabolic environment characterizing it as an ideal form for delivery [189]. Until now, LNA-anti-miR-221 has been used in a preclinical xenograft model and showed a significant antitumor effect [190]. miRNA sponges are enclosed in the vector and, when delivered, act as a competitor for the binding site of mRNA of pro-oncogenic miRNA [191]. miRNA sponges have been used to inhibit the progression of metastasis in breasts where the pro-oncogenic effect of miR-9 is masked via the amelioration of miR-9 binding site’s interaction with the mRNA. Apart from the aforementioned immuno-therapeutics of miRNA in cancer, miRNA antagonists are also extensively used, as this class of miRNA antagonizes the pro-oncogenic effect of miRNA by interacting with the various immunogenic cells.

Furthermore, miRNA-155 is known to increase the expression of PD-L-1 and its antagonist (MRG105) has been reported to ameliorate tumour growth [192]. In addition to miR-155, miR-23a, which is released under the influence of TGF-β (secreted from tumors), has been reported to diminish the cytotoxic potential of CD8+ cells antagonist, and exhibited a potent antitumor effect against melanoma [178]. Additionally, the use of miR-17-92 clusters in combination with chimeric antigen cells and in combination with temozolomide has increased the therapeutic efficacy and survival rate among patients of glioblastoma [192].

## 8. Conclusions

Based on the discussion, it seems persuasive that miRNA acts as potent modulators of carcinogenesis. Numerous published studies present concrete evidence for their immunotherapeutic implication against various types of cancers [24]. Furthermore, miRNA is not only implicated in cancer immunotherapy, but it also plays a pivotal role in the diagnosis and prognosis of cancer [193]. This is achieved by either determining the specific profile of miRNA within the immune cells or by analyzing the miRNA derived from the micro-vesicles of tumour patients [193]. However, further intensive research is needed to develop a diagnostic approach and to transport such a concept from bench to bedside. Moreover, some of the shortcomings of miRNA-based therapeutic interventions must be considered. A high level of inconsistencies in the formulation of miRNA and its delivery system is observed [85]. We herein speculate that the standard pharmacological preparations and precise pharmacodynamic–pharmacokinetics will control the toxic effect of these miRNA-based therapeutics [85]. Furthermore, based on ample published evidence, the development of miRNA-based immune therapeutics and to reduce immune escape has been attempted.

Additionally, we also observed that most of the preclinical study was conducted using an acute dose regimen, and thus, the effects observed via long-term (chronic) study need to be explored in detail. It is also important to discuss that despite tremendous ongoing preclinical and clinical work to develop miRNA-based immunotherapy satisfactory clinical outcome in cancer has not yet been achieved. For example, MRX34 showed a potent anticancer effect in the preclinical study, but immunogenic severe toxicity was observed when tested in cancer patients. However, this unexpected outcome confirmed that the miRNA could cause unwanted non-specific immune-related responses [194,195]. RNA interference (RNAi) causes TLR signalling pathway activation and interferes with the speculated therapeutic outcome [196]. To cater to these issues, a strategy such as combinatorial use of miRNA mimic and siRNA or miRNA along with chemotherapy can be used to mitigate the immune complication and other toxicities [192,197]. Apart from the combinatorial approach, various nanoparticle-based miRNA, also known as ‘miRNome’ and novel drug delivery systems (nanotubes and liposome), have been developed to offer additional advantages over conventional miRNA of safety, efficacy, and stability [198]. This approach also possesses benefits and constraints. The benefits include the fact that nanoparticles can be produced rapidly, drug loading capacity can be modulated, a controlled amount of miRNA can be delivered, and targeted delivery can be achieved [198]. However, some of the constraints for using nanoparticles as a carrier of miRNA include a low efficacy in transfusion, the requirement of multiple administrations, and an increased risk of immunological toxicity [199]. Therefore, we conclude that miRNA-based immunotherapy is a novel and emerging therapeutic method for the treatment and management of cancer, but more extensive studies are required for the safe and effective targeted delivery of miRNA in cancer patients.

## Figures and Tables

**Table 1:** Examples of oncogenic miRNAs.

S. No.	Type of Cancer	miRNAs Involved		References
1	Acute Myeloid Leukemia	miR-196b; miR-126; miR-9; miR-17-92	Oncogenic	[59]
2	Gastric	miR-421; miR-196a	Oncogenic	[56,57]
3	Breast	miR-181a/b, miR-21 miR-34, miR-34 b/c	Oncogenic Tumor Suppressor	[60,61]
4	Non-Small Cell Lung Cancer	miR-27a	Oncogenic	[62]
5	Renal Cell Carcinomas	miR-30a/c	Oncogenic	[63,64]
6	Colorectal	miR-34a, miR-34b, miR34b/c	Tumor suppressor	[65,66,67]
7	Prostate	miR-34a, miR-34b, miR34b/c	Tumor suppressor	[68,69]
8	Lung	miR-34a, miR-34b, miR34b/c	Tumor Suppressor	[70,71,72]
9	Liver	miR-34b	Tumor Suppressor	[73]
10	Ostosarcoma	miR-34a, miR34b, miR-192	Tumor Suppressor	[74,75,76]
11	haematological neoplasms	miR-34a, miR-34b-5p, miR-34c, miR-34b/c	Tumor Suppressor	[77,78,79]
12	Lymphophytic leukemia	miR-15/16	Tumor Suppressor	[80]

**Table 2:** miRNAs biomarkers and their role in various types of cancer [11,95,99,107].

Sl. No.	Type of Cancer	miRNAs Biomarkers		
		Predictive	Prognostic	Diagnostic
1	Prostate cancer	miR-21	miR-20a; miR -21; miR-141	miR-141, -375; let-7c, -7e miR-141
2	Lung cancer	miR-128b	miR-221; let-7a, -137; miR-372; miR -182; miR-15b, -16	miR-16, miR-17, -19b, miR-200 family; miR-29c, -30c
3	Breast cancer	miR-125b	miR-210; miR-10b	miR-21, -30a; miR-141, -145; miR- 801
4	Ovarian cancer	miR-181a,b, -213; miR-23a, -27a; let-7g, 3p	miR-200 family; miR-410, -645	miR-126, -127; miR-200 & let-7 family; miR-21, -29a
5	Liver cancer	miR-21, -200b	miR-200 family; miR-21, -22, -26	miR-200c, -203, -224; miR-222, -223

**Table 3:** Role of miRNAs in Cancer immune escape [127].

miRNA	Effect/Mechanism	Cancer Types
miR-24-3p	Increased T reg cells activity and reduced Th17 proliferation via targeting fibroblast growth factor 11	Nasopharyngeal cancer
miR-183	Reduced the activity of NK cells	Lung cancer
miR-23a	Reduced activity of CD8+ cells via acting on B lymphocyte-induced maturation protein-1	
miR-155	Reduced activity of MDSCs via acing on hypoxia inducing factor-α	pancreatic cancer
miR-212-3p	Increased immune response of DC via acting on Regulatory Factor X Associated Protein	
miR-92a-3p	Increased tumor-associated macrophages and IL-6 level	Liposarcoma
miR-25-3p	Increased tumor-associated macrophages and IL-6 level	
miR-155	Reduced activity of MDSCs via acing on hypoxia inducing factor-α	Skin cancer
miR-210	Reduced activity of MDSCs via acing on C-X-C motif chemokine 12 and IL-16	
miR-34a	Reduced recruitment of T reg cells via acting on C-X-C motif chemokine 12	Hepatic cancer
miR-20a and miR-17-5p	Reduced activity of MDSCs via acing on Reduced activity of MDSCs via acing on C-X-C motif chemokine 12 and IL-16	Colon cancer
miR-494	Reduced activity of MDSCs via acing on Phosphatase and tensin homolog	Breast cancer
miR-222-3p	Increased polarization of M2 macrophage via acting on suppressor of cytokine signaling 3	Ovarian cancer

**Table 4:** Showing the modulatory effect of miRNAs via immune cells on tumorigenesis.

miRNA	Immune Cells/Cancer Cells	Effect	Cancer Types	References
miR-19a-3p	Macrophages (M2)	Polarization of macrophage and amelioration of cancer progression and metastasis	Breast cancer	[162]
miR-21	T reg cells	Reduce the proliferation of T reg cells leading to reduced survival and proliferation		[163]
miR-23a/27a/24-2	Macrophage	Polarization of M2 macrophage and promote tumour growth		[164]
miR-126	Cancerous cells	Reduced monocyte recruitment and stimulated metastasis		[165]
miR-146a	Macrophages	Activation of NF-kB and promote tumour cell invasion		[140]
miR-155	Cancerous cells	Activation of JAK-STAT pathway and proliferation of tumor cells.		[166]
miR-223	Macrophages (M2)	Differentiation of macrophage and stimulate tumour cells invasion		[88]
miR-494	MDSCs	MDSCs accumulation stimulate tumor cells invasion and metastasis		[167]
miR-20a	Cancerous cells	Suppress NK-mediated antitumor effect and promote tumour invasion as well as proliferation	Ovarian cancer	[168]
miR-424	Cancerous cells	Activate T cells and stimulate sensitivity of tucells towards chemotherapy		[169]
miR-199a	Cancerous cells	Production of cytokines and progression of tumorigenesis		[170]
miR-34a/c	Cancerous cells	Suppress NK-mediated antitumor effect and promote tumour invasion as well as proliferation	Skin cancer	[171]
miR-17	T cells	Alters the function of T cells and promote tumour growth		[172]
miR-155	MDSCs	Increased HIF-α and promote tumor growth		[173]
miR-29	Cancer cells	Mitigate the NK cell’s function, T cells and promote the growth of tumors	Solid tumors	[174]
miR-214	CD4+ and CD25+ T cells	Stimulate T reg cells and promote tumour growth		[175]
miR-146a	Cancer cells	Reduced IL-8, TRAF-6 and exhibit the antitumor effect	Gastric cancer	[176]
miR-5, 18 and 22	Cancer cells	Reduced PD-1 expression 6 and exhibit antitumor effect		[177]
miR-23a	T cells	Inhibit CD8+ function and promote tumour growth and TGF-β induced tumour invasion	Lung cancer	[178]
miR-155	Dendritic cell	Stimulate dendritic cell maturation and activation of T cells		[179]
miR-124	T cells	Inhibit STAT-3 and promote T cell-induced killing of tumour cells	Glioblastoma	[180]
miR-15a and 16a	T cells	Reduced PD-1 and increased CD8+ mediated antitumor effect		[181]
miR-28	T cells	Enervation of T cells and reduced PD-1		[182]
miR-138	T cells	Diminished expression of PD-1 and exhibit the anti-tumor effect		[183]
miR-182	NK cells	Stimulate the tumour-killing potential of NK cells and increase the release of perforin-1	Hepatic cancer	[184]

## Data Availability

No new data were created or analyzed in this review. Data sharing is not applicable to this article.
